# Second primary neoplasms following non-Hodgkin's lymphoma in New South Wales, Australia

**DOI:** 10.1054/bjoc.1999.1102

**Published:** 2000-03-06

**Authors:** P Brennan, M Coates, B Armstrong, D Colin, P Boffetta

**Affiliations:** International Agency for Research on Cancer, 150 cours Albert Thomas, Lyon, 69372, France; New South Wales Central Cancer Registry, 153 Dowling St, Woolloomooloo, New South Wales, Australia

**Keywords:** NHL, ultraviolet radiation, second cancers

## Abstract

The incidence of non-Hodgkin's lymphoma (NHL) has been increasing rapidly over the last three decades. The reasons for this trend are not known although increasing exposure to sunlight has been postulated. We used data from the New South Wales Central Cancer Registry to analyse second primary neoplasms following NHL diagnosed between 1972 and 1995, to identify possible common causal agents. A total of 12 452 patients contributed 54 308 person-years of follow-up during which time there were 705 second primary neoplasms compared to 592.99 expected (standardized incidence ratio (SIR = 1.19, 95% confidence interval (CI) 1.10–1.28). There were excesses of melanomas of skin (SIR = 2.38, 95% CI 1.92–2.91), lip cancer (SIR = 2.74, 95% CI 1.59–4.38), tongue cancer (SIR = 2.53, 95% CI 1.09–4.99) and bladder cancer (SIR = 1.64, 95% CI 1.19–2.21). There was also over a threefold excess in soft tissue sarcomas (SIR = 3.61, 95% CI 1.80–6.45) and in thyroid cancer (SIR = 3.42, 95% CI 1.56–6.49). The SIR for myeloid leukaemia was 0.78 (95% CI 0.28–1.69). The increases in melanoma of the skin and cancer of the lip and tongue among patients with NHL strongly suggest sunlight exposure as a shared causal agent. The increase in soft tissue sarcomas might be due to shared effects of exposure to chemicals such as phenoxy acid herbicides. The increases in bladder and thyroid cancers are likely to be explained by effects of treatment for NHL. We did not find a chemotherapy related increased risk of myeloid leukaemia among NHL patients. © 2000 Cancer Research Campaign


*British Journal of Cancer* (2000) **82**(7), 1344–1347
© 2000 Cancer Research Campaign
DOI: 10.1054/ bjoc.1999.1102, available online at http://www.idealibrary.com on IDEAL®

### Second primary neoplasms following non-Hodgkin's lymphoma in New South Wales, Australia

**P Brennan[1], M Coates[2], B Armstrong[2], D Colin[1] and P Boffetta[1]**

[1]International Agency for Research on Cancer, 150 cours Albert Thomas, Lyon 69372, France; [2]New South Wales Central Cancer Registry, 153 Dowling St, Woolloomooloo, New South Wales, Australia

**Summary** The incidence of non-Hodgkin's lymphoma (NHL) has been increasing rapidly over the last three decades. The reasons for this trend are not known although increasing exposure to sunlight has been postulated. We used data from the New South Wales Central Cancer Registry to analyse second primary neoplasms following NHL diagnosed between 1972 and 1995, to identify possible common causal agents. A total of 12 452 patients contributed 54 308 person-years of follow-up during which time there were 705 second primary neoplasms compared to 592.99 expected (standardized incidence ratio (SIR = 1.19, 95% confidence interval (CI) 1.10–1.28). There were excesses of melanomas of skin (SIR = 2.38, 95% CI 1.92–2.91), lip cancer (SIR = 2.74, 95% CI 1.59–4.38), tongue cancer (SIR = 2.53, 95% CI 1.09–4.99) and bladder cancer (SIR = 1.64, 95% CI 1.19–2.21). There was also over a threefold excess in soft tissue sarcomas (SIR = 3.61, 95% CI 1.80–6.45) and in thyroid cancer (SIR = 3.42, 95% CI 1.56–6.49). The SIR for myeloid leukaemia was 0.78 (95% CI 0.28–1.69). The increases in melanoma of the skin and cancer of the lip and tongue among patients with NHL strongly suggest sunlight exposure as a shared causal agent. The increase in soft tissue sarcomas might be due to shared effects of exposure to chemicals such as phenoxy acid herbicides. The increases in bladder and thyroid cancers are likely to be explained by effects of treatment for NHL. We did not find a chemotherapy related increased risk of myeloid leukaemia among NHL patients. © 2000 Cancer Research Campaign

**Keywords:** NHL; ultraviolet radiation; second cancers

The incidence of non-Hodgkin's lymphoma (NHL) is increasing in most parts of the world. A comparison of cancer registry information between 1982 and 1992 (Parkin et al, 1992, 1997) indicates that this increase is occurring at an average annual rate of 4–5% each year, implying a doubling of NHL incidence every 20 years. This upward trend is observed in all geographical regions covered by cancer registration, and is not restricted to any particular age group or sex, or to predominantly rural or urban areas. The reason for the increase has attracted much speculation but there is no clear explanation for it.

The changing diagnostic classification of NHL cannot explain recent increases that have occurred (Cartwright, 1992), while AIDS-related lymphomas represent only a small proportion of all lymphomas in most regions (Rolland-Portal et al, 1997). Other explanations offered for the observed trends in NHL include increasing population exposure to pesticides, organic solvents and hair dyes, but none of these is well supported by the evidence.

Recent interest has focused attention on solar ultraviolet radiation as a possible cause of the increasing incidence for both melanoma of the skin and NHL (Cartwright et al, 1994; McMichael and Giles, 1996). To investigate this hypothesis further we have studied second primary cancers in NHL patients registered with the New South Wales (NSW) Central Cancer Registry in Australia. This registry was considered to be particularly suitable because of its size and period of time in operation (24 years), and because NHL and melanoma incidence rates in New South Wales are among the highest in the world (Parkin et al, 1997). Our a priori hypothesis was that if sunlight was related to the aetiology of NHL we would expect an excess of melanoma of the skin among patients with NHL.

*Received 14 July 1999*
*Revised 20 September 1999*
*Accepted 20 September 1999*

*Correspondence to:* P Brennan

### METHODS

The NSW Central Cancer Registry is a population-based registry which first reported cancer statistics for the year 1972. The registry covers the population of NSW of over 6 000 000, of whom approximately two-thirds live in the cities of Sydney, Newcastle and Wollongong. Approximately 25% of the population are migrants to Australia. The prime source of cancer data is the compulsory notification form completed by hospital staff for each cancer case in every public and private hospital. In addition, each department of radiation oncology must notify each cancer case when first treated in the department. Pathology laboratories must also send copies of all reports of a diagnosis of cancer. Each person is given a unique identification number when first registered. Subsequent primary cancers are registered and linked to the first through the identification number. Tumour site was coded according to the International Classification of Disease, Ninth Revision (ICD-9). Population census data were provided by the Australian Bureau of Statistics which publishes annual estimates of the mid-year resident population based on a quinquennial census.

From 1972 to 1995 a total of 15 330 NHL patients (ICD-9 codes 200, 202) were registered in NSW. To avoid detection bias in cancers present at the time of NHL diagnosis, the follow-up period did not start until 3 months after NHL diagnosis. A total of 2878 cases either died or were reported as having a second primary

**Table 1** Characteristics of NHL patients, New South Wales, 1972–1995

| | Free of second neoplasm ($n$ = 11 747) | Second neoplasm ($n$ = 705) |
|---|---|---|
| Sex | | |
| Male | 6297 (54%) | 427 (61%) |
| Female | 5450 (46%) | 278 (39%) |
| Age at diagnosis of NHL | | |
| < 51 | 2979 (25%) | 88 (12%) |
| 51–64 | 3384 (29%) | 274 (39%) |
| 65–70 | 1922 (16%) | 134 (19%) |
| 71+ | 3462 (30%) | 209 (30%) |
| Year of diagnosis of NHL | | |
| Before 1980 | 2393 (20%) | 201 (29%) |
| 1980–1984 | 2189 (19%) | 182 (26%) |
| 1985–1989 | 2899 (25%) | 213 (30%) |
| After 1990 | 4266 (36%) | 109 (15%) |
| Country of birth | | |
| Oceania | 9004 (77%) | 534 (76%) |
| North/West Europe | 1192 (10%) | 80 (11%) |
| Mediterranean Europe | 452 (4%) | 26 (4%) |
| Central/East Europe | 339 (3%) | 35 (5%) |
| Other/Unknown | 760 (6%) | 30 (4%) |
| Died during follow-up | 7156 (61%) | 515 (73%) |

neoplasm within 3 months of the diagnosis of NHL. Exclusion of these left a study population of 12 452 patients. For each patient the length of time at risk of a subsequent cancer was calculated from 3 months after the date of NHL diagnosis to the first of (i) the date of diagnosis of second neoplasm, (ii) date of death, or (iii) the end of the study follow-up (31 December 1995), whichever was earliest. Mortality follow-up was conducted through linkage to the NSW register of deaths and the Australian National Death Index.

To assess any possible excess of second primary neoplasms in the cohort of primary NHL, we compared the observed number of neoplasms to the expected number derived from the age- and sex-specific cancer incidence rates of the NSW population for the years 1972–1995, after excluding the expected number of NHL Standardized incidence ratios (SIR) adjusted for age, year, sex and country of birth were calculated using indirect standardization methods. Exact confidence intervals (CI) around the SIR were calculated assuming a Poisson distribution for the observed number of neoplasms. This analysis was conducted for all neoplasms combined and also for specific cancer sites. The NSW Cancer Registry does not routinely register non-melanocytic skin cancers.

### RESULTS

The 12 452 patients contributed 54 308 person-years of follow-up time during which there occurred 705 second primary neoplasms. As might be expected, those with a second neoplasm were more likely to be male, to be older and to be diagnosed earlier in the study period than those who were free of a second neoplasm (Table 1). The two groups were comparable with respect to their countries of origin, with approximately 75% being born in Oceania (mainly Australia and New Zealand). The next largest group comprised immigrants from northern and western Europe, the majority of whom were from the UK and Ireland. Nearly all those who had migrated from the Mediterranean region had been born in Italy, Greece or Malta. The majority of patients from Central/Eastern Europe were from Poland, Hungary and former-Yugoslavia.

The observed 705 second neoplasms in NHL patients were compared to the number that would have been expected given the demographic structure of the study population and the local cancer incidence rates during the study period (Table 2). The expected number of second neoplasms was 592.99 (SIR = 1.19, 95% CI 1.10–1.28). This 19% increase in the observed cancer incidence was mainly due to over a twofold excess of melanoma of the skin based on 93 cases (SIR = 2.38, 95% CI 1.92–2.91). However, a significant ($P < 0.05$) increase in risk of a cancer was also observed at five other sites. There was over a twofold increase in cancer of the lip based on 17 observed cases (SIR = 2.74, 95% CI 1.59–4.38), and a similar increase in tongue cancer based on eight cases (SIR = 2.53, 95% CI 1.09–4.99). There was over a threefold excess in soft tissue sarcomas based on 11 cases (SIR = 3.61, 95% CI 1.80–6.45), and a 64% excess of bladder cancer was observed based on 44 cases (SIR = 1.64, 95% CI 1.19–2.21). Also, there was a sizeable increase in thyroid cancer based on nine cases (SIR = 3.42, 95% CI 1.56–6.49). The observed and expected numbers of the remaining cancer sites were all roughly similar to each other. A 25% excess of colon cancer based on 80 cases (SIR = 1.25, 95% CI 0.99–1.55), and a deficit of pancreatic cancer based on eight cases (SIR = 0.45, 95% CI 0.20–0.90) may be due to chance given that over 30 comparisons were made.

The six cancer sites that were observed in excess were analysed in more detail after stratifying for sex, age, year of diagnosis, time period after NHL diagnosis and geographic region of birth (Table 3). The excess of melanoma of the skin was observed equally among men and women, over all age groups, over the whole of the study period, and also in all time periods after diagnosis of NHL. However, it was restricted to those born in Oceania and immigrants from North/West Europe and was not found among immigrants from Mediterranean Europe or Central/Eastern Europe (expected 2.7 cases). Stratified analysis for the other five cancer sites did not indicate any strong trends although numbers were small.

### DISCUSSION

Our analysis of more than 12 000 primary NHL, and over 700 subsequent second neoplasms, revealed a 19% increased risk of all second cancers combined, which was due to increased risks for melanoma of the skin and cancers of the lip, tongue, bladder and thyroid, and also soft tissue sarcomas.

A systematic review of all previous studies investigating second cancers after NHL has recently been conducted (Boffetta et al, 1999). This indicated that NHL patients experienced large excess risks of acute non-lymphocytic leukaemia (ANLL), Hodgkin's disease and melanoma of the skin, and smaller increases for lung, kidney and bladder cancers. The risk of ANLL and bladder cancer was considered to be related to chemotherapy, especially use of cyclophosphamide for bladder cancer (Travis et al, 1995), and use of prednimustine and mechlorethamine or procarbazine for ANLL (Travis et al, 1994). Radiotherapy use was associated with risk of both ANLL and lung cancer. Melanoma of the skin was not associated with NHL treatment.

Similar to previous reports, the observed increase in bladder cancer in our study is likely to be due to initial treatment of the NHL with cyclophosphamide. Cyclophosphamide was used consistently in combination chemotherapy for NHL in Australia over the period of diagnosis of the cases. Also, given the known epidemiology of thyroid cancer, the observed excess at this site is

© 2000 Cancer Research Campaign

**Table 2** Observed and expected numbers of second primary cancers following a diagnosis of NHL, New South Wales, 1972–1995

| Site (ICD9 code) | Observed | Expected | SIR | 95% confidence interval |
|---|---|---|---|---|
| **All neoplasms** | **705** | **592.99** | **1.19** | **(1.10–1.28)** |
| **Lip (140)** | **17** | **6.21** | **2.74** | **(1.59–4.38)** |
| **Tongue (141)** | **8** | **3.16** | **2.53** | **(1.09–4.99)** |
| Salivary gland (142) | 4 | 1.80 | 2.22 | (0.60–5.70) |
| Mouth (143–145) | 5 | 4.04 | 1.24 | (0.40–2.89) |
| Pharynx (146–149) | 6 | 4.68 | 1.28 | (0.47–2.79) |
| Oesophagus (150) | 13 | 8.9 | 1.45 | (0.77–2.48) |
| Stomach (151) | 18 | 23.00 | 0.78 | (0.46–1.24) |
| Colon (153) | 80 | 64.21 | 1.25 | (0.99–1.55) |
| Rectum (154) | 38 | 33.85 | 1.12 | (0.79–1.54) |
| Liver (155) | 4 | 2.95 | 1.35 | (0.36–3.47) |
| Gallbladder (156) | 4 | 5.57 | 0.72 | (0.19–1.84) |
| **Pancreas (157)** | **8** | **17.59** | **0.45** | **(0.20–0.90)** |
| Nose, sinuses (160) | 1 | 1.06 | 0.94 | (0.01–5.25) |
| Larynx (161) | 5 | 6.83 | 0.73 | (0.24–1.71) |
| Lung (162) | 83 | 82.36 | 1.01 | (0.80–1.25) |
| Bone (170) | 2 | 0.80 | 2.51 | (0.30–9.04) |
| **Soft tissue sarcoma (171)** | **11** | **3.05** | **3.61** | **(1.80–6.45)** |
| **Melanoma of skin (172)** | **93** | **39.13** | **2.38** | **(1.92–2.91)** |
| Female breast (174) | 46 | 51.10 | 0.90 | (0.66–1.20) |
| Cervix uteri (180) | 4 | 5.88 | 0.68 | (0.18–1.74) |
| Corpus uteri (182) | 9 | 8.52 | 1.06 | (0.48–2.00) |
| Ovary (183) | 9 | 7.74 | 1.16 | (0.53–2.21) |
| Prostate (185) | 84 | 81.98 | 1.02 | (0.82–1.27) |
| Testis (186) | 0 | 0.90 | 0 | (0.00–4.10) |
| **Bladder (188)** | **44** | **26.77** | **1.64** | **(1.19–2.21)** |
| Kidney (189) | 20 | 16.11 | 1.24 | (0.76–1.92) |
| Brain, nervous stemem (191–192) | 6 | 7.94 | 0.76 | (0.28–1.64) |
| **Thyroid (193)** | **9** | **2.63** | **3.42** | **(1.56–6.49)** |
| Hodgkin's disease (201) | 2 | 1.67 | 1.20 | (0.13–4.32) |
| Multiple myeloma (203) | 5 | 7.96 | 0.63 | (0.20–1.47) |
| Lymphoid leukaemia (204) | 5 | 6.15 | 0.81 | (0.26–1.90) |
| Myeloid leukaemia (205) | 6 | 7.71 | 0.78 | (0.28–1.69) |
| Other leukaemia (206–208) | 3 | 2.39 | 1.26 | (0.26–3.67) |
| Others | 53 | 48.35 | 1.10 | (0.82–1.43) |

**Sites in bold** represent a statistically significant (*P* < 0.05) excess or deficit from the expected value.

**Table 3** Observed and SIR for second primary neoplasms following NHL, New South Wales, 1972–1995, stratified by demographic characteristics

| | Lip cancer | | | Tongue cancer | | | Soft tissue sarcoma | | | Melanoma of skin | | | Bladder cancer | | | Thyroid cancer | | |
|---|---|---|---|---|---|---|---|---|---|---|---|---|---|---|---|---|---|---|
| | O | SIR | 95% CI | O | SIR | 95% CI | O | SIR | 95% CI | O | SIR | 95% CI | O | SIR | 95% CI | O | SIR | 95% CI |
| Sex | | | | | | | | | | | | | | | | | | |
| Men | 10 | 2.08 | (1.0–3.8) | 3 | 1.43 | (0.3–4.2) | 9 | 4.81 | (2.2–9.1) | 60 | 2.41 | (1.8–3.1) | 36 | 1.74 | (1.2–2.4) | 2 | 2.04 | (0.2–7.4) |
| Women | 7 | 4.95 | (2.0–10) | 5 | 4.72 | (1.5–11) | 2 | 1.70 | (0.2–6.1) | 33 | 2.31 | (1.6–3.2) | 8 | 1.31 | (0.6–2.6) | 7 | 4.24 | (1.7–8.8) |
| Age at NHL | | | | | | | | | | | | | | | | | | |
| diagnosis <51 | 2 | 2.52 | (0.3–9.1) | 0 | 0 | (0.0–8.6) | 1 | 2.50 | (0.1–14) | 15 | 2.14 | (1.2–3.5) | 4 | 2.73 | (0.7–7.0) | 3 | 4.35 | (0.9–12) |
| 51–64 | 7 | 3.45 | (1.4–7.1) | 5 | 3.82 | (1.2–8.9) | 4 | 4.28 | (1.1–11) | 41 | 2.92 | (2.1–4.0) | 12 | 1.47 | (0.8–2.6) | 4 | 4.60 | (1.2–11) |
| 65–70 | 4 | 3.00 | (0.8–7.7) | 0 | 0 | (0.0–5.5) | 3 | 4.56 | (0.9–13) | 19 | 2.41 | (1.5–3.8) | 11 | 1.66 | (0.8–3.0) | 0 | 0 | (0.0–8.2) |
| 71+ | 4 | 2.0 | (0.5–5.0) | 3 | 3.98 | (0.8–12) | 3 | 2.83 | (0.6–8.3) | 18 | 1.76 | (1.0–2.8) | 17 | 1.62 | (0.9–2.6) | 2 | 3.24 | (0.4–12) |
| Year of diagnosis | | | | | | | | | | | | | | | | | | |
| 1972–1979 | 3 | 1.50 | (0.3–4.4) | 2 | 1.93 | (0.2–7.0) | 2 | 2.11 | (0.2–7.6) | 26 | 2.39 | (1.6–3.5) | 15 | 1.56 | (0.9–2.6) | 4 | 4.83 | (1.3–12) |
| 1980–1984 | 4 | 2.72 | (0.7–7.0) | 3 | 3.76 | (0.8–11) | 2 | 2.84 | (0.3–10) | 27 | 2.77 | (1.8–4.0) | 11 | 1.61 | (0.8–2.9) | 2 | 3.07 | (0.3–11) |
| 1985–1989 | 7 | 4.24 | (1.7–8.7) | 2 | 2.44 | (0.3–8.8) | 5 | 5.96 | (1.9–14) | 25 | 2.22 | (1.4–3.3) | 11 | 1.73 | (0.9–3.1) | 2 | 2.83 | (0.3–10) |
| 1990–1995 | 3 | 2.73 | (0.6–8.0) | 1 | 1.96 | (0.3–11) | 2 | 3.56 | (0.4–13) | 15 | 2.07 | (1.2–3.4) | 7 | 1.76 | (0.7–3.6) | 1 | 2.25 | (0.1–12) |
| Time period after | | | | | | | | | | | | | | | | | | |
| NHL diagnosis | | | | | | | | | | | | | | | | | | |
| 4–12 months | 1 | 1.14 | (0.1–6.3) | 1 | 2.19 | (0.0–12) | 1 | 2.38 | (0.1–13) | 13 | 2.44 | (1.3–4.2) | 5 | 1.30 | (0.4–3.1) | 0 | 0 | (0.0–10) |
| 1–4 years | 10 | 3.77 | (1.8–6.9) | 4 | 2.85 | (0.8–7.3) | 6 | 4.62 | (1.7–10) | 34 | 2.04 | (1.4–2.8) | 21 | 1.82 | (1.1–2.8) | 3 | 2.59 | (0.5–7.6) |
| 5–9 years | 4 | 2.63 | (0.7–6.7) | 2 | 2.51 | (0.3–9.1) | 4 | 5.28 | (1.4–14) | 33 | 3.32 | (2.3–4.7) | 10 | 1.50 | (0.7–2.8) | 2 | 2.99 | (0.3–10) |
| 10+ years | 2 | 1.72 | (0.2–6.2) | 1 | 1.97 | (0.3–11) | 0 | 0 | (0.0–6.7) | 13 | 1.81 | (1.0–3.1) | 8 | 1.68 | (0.7–3.3) | 4 | 9.29 | (2.5–24) |
| Country of origin | | | | | | | | | | | | | | | | | | |
| Oceania | 15 | 3.20 | (1.8–5.3) | 7 | 2.91 | (1.2–6.0) | 8 | 3.44 | (1.5–6.8) | 75 | 2.52 | (2.0–3.2) | 32 | 1.58 | (1.1–2.2) | 9 | 4.44 | (2.0–8.4) |
| North/West Europe | 1 | 1.39 | (0.1–7.7) | 1 | 2.87 | (0.0–16) | 1 | 2.89 | (0.1–16) | 10 | 2.37 | (1.1–4.4) | 7 | 2.15 | (0.9–4.4) | 0 | 0 | (0.0–13) |
| Med Europe | 0 | 0 | (0.0–17) | 0 | 0 | (0.0–31) | 2 | 19.6 | (2.2–71) | 0 | 0 | (0.2–53) | 1 | 1.22 | (0.1–6.8) | 0 | 0 | (0.0–41) |
| Cent/East Europe | 0 | 0 | (0.0–18) | 0 | 0 | (0.0–18) | 0 | 0 | (0.0–37) | 1 | 0.82 | (0.1–4.6) | 3 | 3.2 | (0.6–9.3) | 0 | 0 | (0.0–53) |
| Other* | 1 | 2.64 | (0.1–14) | 0 | 0 | (0.0–19) | 0 | 0 | (0.0–21) | 7 | 2.8 | (1.1–5.6) | 1 | 0.68 | (0.0–3.8) | 0 | 0 | (0.0–23) |

O = Observed, SIR = standardized incidence ratio, Other* = Asia/Middle East/Americas/Africa/Unknown.

 *© 2000 Cancer Research Campaign*

likely to be related to radiotherapy (Ron, 1996). This excess was most apparent 10 years after the initial diagnosis of NHL, and is thus consistent with a late effect of treatment. Interestingly, we did not observe any excess for leukaemia as a whole or myeloid leukaemia in particular as a second cancer, in contrast to the large excesses observed in previous studies (Boffetta et al, 1999). The increase in ANLL observed in other cohorts is assumed to be due to one or more components of MOPP chemotherapy regimes (mechlorethamine, vincristine, procarbazine and prednisone), and possibly radiation therapy (Travis et al, 1991). It is possible that the implementation of treatment regimes for NHL may have been different in New South Wales over the study period compared to the USA. Moreover, the SIR for leukaemia, and for myeloid leukaemia in particular, was close to 1.0 for all calendar periods, and also all time periods after diagnosis of NHL (data not shown). This would also indicate that changes over time in the coding of ANLL cannot explain the absence of an association.

The joint association between NHL and melanoma of the skin has previously been hypothesized to be due to solar ultraviolet radiation (McMichael and Giles, 1996; Cartwright et al, 1994), although another shared cause acting through an immunosuppressive effect is possible. The observed increase in cancer of the lip after NHL, which has not been previously noted, supports the suggestion of a shared effect of sun exposure. Although expected numbers were small, no cases of melanoma of the skin or lip cancer were observed among migrants from Mediterranean Europe, who, due to their darker complexion, are less susceptible to ultraviolet associated cancers than most people born in Australia or Northern or Western Europe (Scotto et al, 1996). These data do however need to be interpreted cautiously as information on country of birth for melanoma of the skin is available on only 47% of cases registered in NSW. The increase in tongue cancer may also be related to sunlight exposure. Although cancer of the tongue is not normally considered to be an ultraviolet related neoplasm, there is over a 1000-fold increase in tongue cancer among patients with xeroderma pigmentosum, a rare inherited cancer-prone DNA repair disorder due to marked hypersensitivity to ultraviolet light (Kraemer et al, 1994). These cancers tend to be restricted to the tip of the tongue. Information on subsite of the tongue in the current study was recorded for only four of the eight cases. Two of these cases were recorded as base of the tongue and two were recorded as tip of the tongue. The increase in soft tissue sarcomas might support the view that NHL and soft tissue sarcomas are both associated with exposure to chemicals such as phenoxy acid herbicides (Kogevinas et al, 1997).

In summary, we have confirmed the association between NHL and melanoma of the skin, which may be explained by joint exposure to solar ultraviolet radiation. We have also identified an excess of lip and tongue cancer after NHL which might be explained by the same relationship. Our results provide support for sunlight exposure as a cause of the increasing incidence of NHL.

### ACKNOWLEDGEMENT

The New South Wales Central Cancer Registry is funded by the New South Wales Health Department and managed by the New South Wales Cancer Council.

### REFERENCES

Boffetta P, Butler J, Maynadie M and Brennan P (1999) Lymphomas, In: *Multiple Primary Cancers*, Neuget AT, Robinson E, Meadows (eds). Lippincott Williams and Wilkins, Philadelphia

Cartwright RA (1992) Changes in the descriptive epidemiology of non-Hodgkin's lymphoma in Great Britian? *Cancer Res* **52**: 5441s–5442s

Cartwright R, McNally R and Staines A (1994) The increasing incidence of non-Hodgkin's lymphoma (NHL): the possible role of sunlight. *Leuk Lymph* **14**: 387–394

Kogevinas M, Becher H, Benn T, Bertazzi PA, Boffetta P, Bueno-de-Mesquita HB, Coggon D, Colin D, Flesch-Janys D, Fingerhut M, Green L, Kauppinen T, Littorin M, Lynge E, Mathews JD, Neuberger M, Pearce N and Saracci R (1997) Cancer mortality in workers exposed to phenoxy herbicides, chlorophenols, and dioxins. An expanded and updated international cohort study. *Am J Epidemiol* **145**: 1061–1075

Kraemer KH, Lee MM, Andrews AD and Lambert WC (1994) The role of sunlight and DNA repair in melanoma and nonmelanoma skin cancer. The xeroderma pigmentosum paradigm. *Arch Dermatol* **130**: 1018–1021

McMichael AJ and Giles GG (1996) Have increases in solar ultraviolet exposure contributed to the rise in incidence of non-Hodgkin's lymphoma? *Br J Cancer* **73**: 945–950

Parkin DM, Muir CS, Whelan SL, Gao YT, Ferlay J and Powell J (eds) (1992) *Cancer Incidence in Five Continents, Vol. VI*. IARC Scientific Publications No. 120, IARC: Lyon

Parkin DM, Whelan SL, Ferlay J, Raymond L and Young J (eds) (1997) *Cancer Incidence in Five Continents, Vol. VII*. IARC Scientific Publications No. 143. IARC: Lyon

Rolland-Portal I, Tazi MA, Milan C, Couillault C and Carli PM (1997) Non-Hodgkin's lymphoma: time trends for incidence and survival in Cote-d'Or, France. *Int J Epidemiol* **26**: 945–952

Ron E (1996) Thyroid cancer. In: Cancer epidemiology and prevention, 2nd edn, Schottenfeld D and Fraumeni J (eds) pp 1000–1021. Oxford University Press: New York

Scotto J, Fears TR and Fraumeni JR (1996) Solar radiation. In: Cancer epidemiology and prevention, 2nd edn, Schottenfeld D and Fraumeni JR (eds) pp 355–372. Oxford University Press: New York

Travis LB, Curtis RE, Boice JD Jn, Hankey BF and Fraumeni JF Jr (1991) Second cancers following non-Hodgkin's lymphoma. *Cancer* **67**: 2002–2009

Travis LB, Curtis RE, Stovall M, Holowaty EJ, van Leeuwen FE, Glimelius B, Lynch CF, Hagenbeek A, Li CY, Banks PM et al (1994) Risk of leukemia following treatment for non-Hodgkin's lymphoma. *J Natl Cancer Inst* **86**: 1450–1457

Travis LB, Curtis RE, Glimelius B, Holowaty EJ, van Leeuwen FE, Lynch CF, Hagenbeek A, Stovall M, Banks PM, Adami J, et al (1995) Bladder and kidney cancers following cyclophosphamide therapy for non-Hodgkin's lymphoma. *J Natl Cancer Inst* **87**: 524–530

© 2000 Cancer Research Campaign
